# The Role of Selenium Mineral Trace Element in Exercise: Antioxidant Defense System, Muscle Performance, Hormone Response, and Athletic Performance. A Systematic Review

**DOI:** 10.3390/nu12061790

**Published:** 2020-06-16

**Authors:** Diego Fernández-Lázaro, Cesar I. Fernandez-Lazaro, Juan Mielgo-Ayuso, Lourdes Jiménez Navascués, Alfredo Córdova Martínez, Jesús Seco-Calvo

**Affiliations:** 1 Department of Cellular Biology, Histology and Pharmacology, Faculty of Health Sciences, University of Valladolid, Campus of Soria, 42003 Soria, Spain; fernandezlazaro@usal.es; 2 Department of Preventive Medicine and Public Health, School of Medicine, University of Navarra, IdiSNA, 31008 Pamplona, Spain; 3 Department of Biochemistry, Molecular Biology and Physiology, Faculty of Health Sciences, University of Valladolid, Campus of Soria, 42003 Soria, Spain; juanfrancisco.mielgo@uva.es (J.M.-A.); a.cordova@bio.uva.es (A.C.M.); 4 Department of Nursing, Faculty of Health Sciences, University of Valladolid, Campus of Soria, 42003 Soria, Spain; lourdes.jimenez@uva.es; 5 Institute of Biomedicine (IBIOMED), Physiotherapy Department, University of Leon, Visiting Researcher of Basque Country University, Campus de Vegazana, 24071 Leon, Spain; dr.seco.jesus@gmail.com

**Keywords:** mineral trace element, selenium, exercise, antioxidants, muscle, hormone response, athletic performance

## Abstract

Exercise overproduces oxygen reactive species (ROS) and eventually exceeds the body’s antioxidant capacity to neutralize them. The ROS produce damaging effects on the cell membrane and contribute to skeletal muscle damage. Selenium (Se), a natural mineral trace element, is an essential component of selenoproteins that plays an important role in antioxidant defense. The activity of the enzyme glutathione peroxidase (GPx), a highly-efficient antioxidant enzyme, is closely dependent on the presence of Se. These properties of Se may be potentially applicable to improve athletic performance and training recovery. We systematically searched for published studies to evaluate the effectiveness of Se supplementation on antioxidant defense system, muscle performance, hormone response, and athletic performance among physically active individuals. We used the Preferred Reporting Elements for Systematic Reviews and Meta-Analysis (PRISMA) guidelines and searched in SCOPUS, Web of Science (WOS), and PubMed databases to identify published studies until March 2020. The systematic review incorporated original studies with randomized controlled crossover or parallel design in which intake of Se administered once a day was compared with the same placebo conditions. No exclusions were applied for the type of physical exercise performed, the sex, nor the age of the participants. Among 150 articles identified in the search, 6 met the criteria and were included in the systematic review. The methodological quality of the studies was evaluated using the McMaster Critical Review Form. Oral Se supplementation with 180 µg/day or 240 µg/day (selenomethionine) and 200 µg/day (Sodium Selenite), significantly decreased lipid hydroperoxide levels and increased GPx in plasma, erythrocyte, and muscle. No significant effects were observed on athletic performance, testosterone hormone levels, creatine kinase activity, and exercise training-induced adaptations on oxidative enzyme activities or on muscle fiber type myosin heavy chain expression. In addition, Se supplementation showed to have a dampening effect on the mitochondria changes in chronic and acute exercise. In summary, the use of Se supplementation has no benefits on aerobic or anaerobic athletic performance but it may prevent Se deficiencies among athletes with high-intensity and high-volume training. Optimal Se plasma levels may be important to minimize chronic exercise-induced oxidative effects and modulate the exercise effect on mitochondrial changes.

## 1. Introduction 

Selenium (Se) is an essential trace element in mammals with antioxidant and immune functions that can be found in seafood, pea lentils, beans, whole grains, organ meats, dairy products, and vegetables [1,2]. Se mineral is a vital element of selenoproteins that are involved in redox catalytic activity, structural, and transport functions. The effects of Se are related to antioxidant defense, synthesis of thyroid hormones, testosterone metabolism, DNA structure, modulation of vitamin E (alpha-tocopherol), anti-carcinogen processes, and muscle performance [2,3]. The primary biological role of Se lies in two fundamental properties: (i) the protective antioxidant function of oxidative damage; and (ii) immunomodulation. These Se properties may potentially be applicable to improve athletic performance and training recovery among physically active individuals [4,5].

During physical exercise, oxygen consumption increases between 10 and 15 times above the resting values and may trigger an elevated production of reactive oxygen species (ROS) [6]. Under physiological conditions, antioxidant systems (enzymatic and non-enzymatic systems) neutralizes the harmful effects of ROS [7]. The selenoprotein family is encoded by 25 genes [8], and two of these genes encode the enzyme glutathione peroxidase (GPx) and the enzyme glutathione reductase (GR). These enzymes comprise the glutathione redox cycle, probably an essential physiological antioxidants system [9]. Glutathione serves as a substrate for GPx to prevent the degradation of cell structures, reducing the action of free radicals and lipid peroxides. GR allows to maintain concentrations of glutathione in the cell, not only to be used by the GPx in the elimination of peroxides but also to detoxify ROS. Glutathione modulates the process of recovery of vitamin C (ascorbic acid) and vitamin E after neutralizing free radicals generated by physical exercise [10]. The GPx/GR antioxidant system is related to different antioxidant systems such as the superoxide dismutase/catalase (SOD/CAT). Both systems, the GPx/GR and the SOD/CAT, do not act simultaneously [11]. The SOD eliminates the superoxide radical before it reacts with susceptible biological molecules or causes other toxic agents [12]. 

In circumstances of elevated consumption of oxygen, such as intense exercise, ROS may exceed the body’s antioxidant capacity to neutralize them [7]. Probably, the risk of cellular injury caused by free radicals may be attenuated by the action of skeletal muscle antioxidant enzymes (GPx, GR, SOD, and CAT). Free radicals can cause lesions in the cell membranes of the skeletal muscle [13]. Some muscle proteins such as creatine kinase (CK), lactate dehydrogenase (LDH), and myoglobin (Mb) have been observed to increase their concentration in the blood after episodes of intense or continuous exercise. In addition, intense exercise may modify the hormonal response, mainly the testosterone/cortisol ratio that regulates the anabolic/catabolic processes involved in protein replenishment [14]. The disruption of normal levels CK, LDH, MB, and testosterone/cortisol ratio are indicative of muscle damage, and may decrease sports performance, and increase the training recovery time, and consequently, may affect the health of athletes [15].

A prevention strategy to avoid the consequence of oxidative stress (OS) and reduce muscle damage may be the oral intake of antioxidant supplementation [7]. The activity of the enzyme glutathione peroxidase (GPx), a highly-efficient antioxidant enzyme, is strictly dependent on the presence of its co-factor Se [16]. Increases in blood levels of Se have been reported through exogenous Se intake, mostly in its organic form of selenomethionine [17] and inorganic salts of sodium selenite (Na2SeO3) [18,19]. Elevated blood concentrations of Se may stimulate the activity of the GSH-Px enzyme in the muscle [11]. The GSH-Px enzyme may protect polyunsaturated fatty acids, proteins, and cell membranes from the effects of peroxides and lipid hydroperoxide (LH), and consequently may lead to prevent exercise-induced muscle damage. Moreover, the GSH-Px participates in the regulation of the inflammatory response [20].

Some studies [21,22,23,24] have examined the impact of antioxidant supplementation on athletes, including minerals’ trace elements with antioxidant properties such as Se [4]. Nevertheless, the properties of Se are not limited to antioxidant activity. Se may play a transcendental biological role as anti-carcinogenic agent and may be related to the reproductive function of humans and the endocrine system. Likewise, Se constitutes a necessary micronutrient for the immune system with a recognized protective role against viral infection. Moreover, elevated serum Se and selenoenzymes (GPx and Se protein) levels have been observed in the early stages of a severe disease characterized by inflammatory response and oxidative stress [25]. However, more studies are needed to confirm the potential properties of Se. 

Furthermore, Se has not been extensively studied in situations involving physical exercise [26]. There is limited information about the effect of Se supplementation and exercise, particularly on its impact on antioxidant systems, muscle damage, hormonal response, and athletic performance. Therefore, the purpose of this study was to critically evaluate the effectiveness of Se supplementation on physiological antioxidant defense system, muscle damage markers, testosterone hormone, and athletic performance in physically active population. In addition, the study aims to determine the effective dose, timing, and duration of treatment for optimal application.

## 2. Material and Methods

### 2.1. Search Strategy

We aimed to systematically review published articles that examined the effects of Se supplementation on exercise performance, physiological indicators of exercise, and antioxidant markers. We followed the Preferred Reporting Elements for Systematic Reviews and Meta-Analysis (PRISMA) guidelines [27], and we structured the PICOS question model for the definition of inclusion criteria as follows: P (population) “healthy exercise practitioners”; I (intervention) “supplementation with selenium”; C (comparison) “same conditions with placebo or control group”; O (outcomes) “antioxidant effect, skeletal muscle status, hormonal response, and athletic performance”, S (study design) “double- or single-blind design and randomized parallel or crossed”.

The systematic review was conducted in Scopus, PubMed, and Web of Science (WOS) databases without language or time restriction. These databases were chosen because they provide high-quality scientific information. The search terms used as search strategy were a combination of Medical Subject Headings (MeSH) and keywords related to Se, skeletal muscle, exercise, antioxidants, and physiology. The search terms included the following: (“athletes” OR “sports people” OR “sports” OR “elite athletes”) AND (“selenium” -Title/Abstract- OR “selenium intake”) AND (“athletic performance” -Title/Abstract- OR “athletic performance/physiology” OR “exercise”) AND (“skeletal muscle” -Title/Abstract-OR “muscle skeletal/physiology”). We used the “snowball method” to screen for additional studies. All titles and abstracts were analyzed meticulously to find any duplicate and were later screened. The full texts of the selected articles were read to verify the inclusion and exclusion criteria were met. This process was conducted by two investigators (D.F.-L. and C.I.F.-L.) that discussed discrepancies.

### 2.2. Selection of Articles: Inclusion and Exclusion Criteria

The inclusion criteria in this systematic review included: (i) human experimental studies involving the supplementation intake of a dose of Se or a product containing Se, before and/or during exercise; (ii) with a control group (lack of Se supplementation intake) in the same experimental conditions (with or without placebo intake); (iii): randomized, parallel or crossover design and double or single-blind studies; (iv) with specific information about the supplemental intake and period of intervention; (v) with information about the type of pharmaceutical form used for the supplementation (pills, tablets, gel caps, liquids); (vi) with at least one reported outcomes related to skeletal muscle status, antioxidant effect, or hormonal response. 

Exclusion criteria included the following: (i) animal studies; (ii) uncontrolled trials; (iii) studies with unknown Se intake dose; (iv) studies conducted among subjects with comorbidities or injuries that prevented the execution of exercise protocols; (v) studies that include Se preparations plus other active ingredients; (vi) editorials, reviews, letters, meeting abstracts, and comments. No inclusion criteria regarding the level of fitness, sex, or age were included. 

We used the McMaster’s Critical Review Form [28] to assess the quality of each of the selected articles. We used this Form to find potential limitations in the methodology of the studies.

The following information was extracted from each study included in the systematic review: name of the first author, year of publication, study design, sample size and characteristics of the included participants such as age, sex, weight, body mass index (BMI), or body fat, dose of supplementation, intervention group, control group, intervention duration, outcomes, and results. Two investigators (D.F.-L. and C.I.F.-L.) conducted the data extraction process using a spreadsheet (Microsoft Inc, Seattle, WA, USA).

## 3. Results

### 3.1. Literature Search

**Figure 1:**
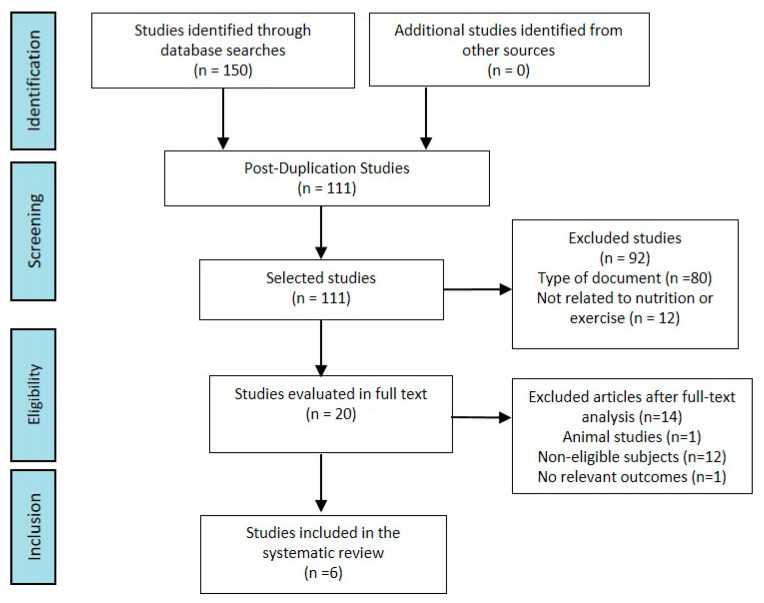
Flow-chart of the literature search and study selection.

The results of the literature search are shown in Figure 1. In total, 150 articles were identified in Scopus, Medline, and WOS until March 2020. After exclusions of duplicates (*n* = 39), 111 records were screened. In the first instance, records were screened by title and abstract content and, 91 articles were excluded. The 20 remaining abstracts were full-text reviewed, and the articles that did not meet the inclusion criteria were excluded. The reasons for the exclusion of studies included: animal studies (*n* = 1), studies among subjects with comorbidities or unable to follow an exercise protocol (*n* = 12), and studies with no relevant outcomes for the purpose of the study (*n* = 1).

### 3.2. Characteristics of the Studies

Among the selected articles, 1 study included active subjects [18], 4 studies regularly trained athletes [13,26,29,30], and 1 study had a population who lacked regular training before the study [19]. Furthermore, in 4 studies, supplementation was organic Se in the form of selenomethionine [13,26,29,30], while in 2 studies, supplementation was in form of salts of sodium selenite [18,19]. Regarding the daily dose of Se, 3 studies used 180 µg [13,26,29], 2 studies used 200 µg [18,19], and 1 study used 240 µg [30]. All studies used a single dose given once a day [13,18,19,26,29,30]. The treatment duration of the studies ranged from 4 to 14 weeks, with 1 study of 4-week duration [18], 1 study of 14 week-duration [19], and 4 studies of 10-duration [13,26,29,30] (Table 1).

### 3.3. Assessment of the Methodological Quality

The results of the assessment of the methodical quality according to the McMaster Quantitative Review Form [28] were as follows: 1 study was evaluated as having “good” quality [13], 4 studies as “very good” [19,26,29,30], and 1 study as “excellent “quality [18]. All studies met the minimum quality score (Table 2).

### 3.4. Findings of Included Studies

A summary of the population, intervention, comparison, outcome measures, and main conclusions is provided in Table 3A–C.

## 4. Discussion

The purpose of this study was to determine the impact of Se supplementation on physiological antioxidant defense systems, muscle damage markers, testosterone hormone, and athletic performance in physically active population. The results suggested that Se oral supplementation of 180 µg/day or 240 µg/day (selenomethionine) and 200 µg/day (sodium selenite) decreased LH levels and significantly increased GPx in plasma, erythrocyte, and muscle, but did not suggest to have an effect on cytochrome C oxidase (Cyt Ox), erythrocyte-reduced glutathione (GSH), SOD, glutathione total (GTtotal), glutathione oxidized (GSSG), erythrocyte glutathione reductase (EGR), vitamin E levels, and succinate dehydrogenase (SDH). In addition, the results did not evidence any improvement in sports performance, testosterone hormone levels, CK activity, oxidative enzyme activities, and the expression of muscle fiber type myosin heavy chain (MHC). Se supplementation was observed to have a dampening effect on the mitochondria changes, including the density of the mitochondria profile, the surface of all the mitochondria profile areas, and the mean surface of individual mitochondria profile areas.

### 4.1. Selenium Supplementation

The Se dietary reference intakes (DRI) for north American adult population have been set by the Institute of Medicine at 55 µg (0.7 µmol)/day, while tolerable upper intake levels (UILs) have been set at 400 µg (5.1 µmol)/day. Se levels higher than the UILs (400 µg/day) causes selenosis disease, which manifests itself as an increased breakability of hair and nails [31]. The dose of Se supplementation administered in the selected studies was 180 or 240 µg/day (selenomethionine) [13,26,29,30] and 200 µg/day (sodium selenite) [18,19], which it is very likely that the total Se intake (dietary + supplementation) of the study population did not exceed tolerable upper intake levels. Moreover, some authors have reported that daily Se intakes of 750 to 850 µg did not produce any adverse effects in adults [32]. No side effects of Se supplementation were reported in any of the studies included in this systematic review. Therefore, it seems that daily doses of Se supplementation of 180 µg/day or 240 µg/day of Se supplementation are apparently safe, as reported previously [33]. For Se supplementation, two pharmaceutical forms of Se preparations were used, organic compounds in the form of selenomethionine [13,26,29,30] and inorganic Se salts in the form of sodium selenite [18,19]. Se supplements in the form of organic compounds (selenomethionine) are generally less likely to be toxic and more bioavailable than inorganic salts [33]. However, both organic [13,26,30] and inorganic [19] Se preparations, significantly increased Se plasm levels when compared with the placebo group. Moreover, both organic and inorganic Se supplementation was effective in maintaining optimal physiological levels [13,19,26,30]. This result may be explained because of the high bioavailability of organic and inorganic Se supplements. The bio bioavailability is substantially higher the bioavailability of other antioxidant supplements such as curcumin, which is administered together with piperine as a bioavailability enhancer [7]. The Se supplementation intake of 180 or 240 µg/day (selenomethionine) and 200 µg/day (sodium selenite) may safely be used as an “enhancer” of the antioxidant defense systems. Although the safety of Se supplementation of the aforementioned dosages has been demonstrated, different characteristics such as the pharmaceutical form of Se, duration of treatment, and exercise modality should be taken into account to adjust the dosage of Se supplementation.

### 4.2. Antioxidant Defense System

The excessive production of ROS exceeds the capacity of neutralization and elimination of the physiological system by altering the homeostatic balance and establishing a state of OS. High concentrations of ROS determine structural modifications in the lipid bilayer of the cellular membranes, nucleic acids, and proteins. In addition, high concentrations of ROS may alter the intracellular signaling pathways by modifying the responses and functions of the cells [34]. The incorporation of Se supplementation may be a practical approach to enhance the antioxidant activity of diets because Se is a more powerful antioxidant than vitamin E, vitamin C, vitamin A, or and Β-carotene. The Se is an important element of the amino acids selenocysteine and selenomethionine, which have antioxidant activity that support the antioxidant enzymatic defence systems [34,35].

Previous studies have evaluated lipid peroxidation by determining LH concentrations in serum [19]. These authors reported that Se supplementation in overweight adults was effective in increasing plasma Se levels near to recommended levels, and decreased the LH responses at rest and after high-intensity exercise. However, in normal-weight individuals, Se supplementation was not effective to reduce LH concentrations. These results may explained because optimal Se levels maximize the expression of GPx activity, but the overweight group had low Se levels before supplementation, and this is the most likely cause of a compromised GPx antioxidant system. The reduction of LH after Se supplementation may be explained by the activation of the GPx system [19]. Although GPx was not measured, it was likely that GPx antioxidant system activity increased because GPx is part of an antioxidant system to protect from the harmful consequences of LH [36] as it has been described by Burk et al. [20].

Se supplementation increased the activity of plasma GPx in some studies [13,26], which suggests that the relationship between GPx and Se may play an important role in the antioxidant GPx defense that detoxifies excess of ROS. Prior research has been demonstrated that exercise upregulates erythrocyte GPx activity [34] and may be enhanced with Se supplementation, as it was previously demonstrated by Tessier et al. [26]. The combination of exercise and Se supplementation may suggest a reinforcement of the antioxidant potential. In muscle, GPx activity was observed to increase with exercise [21]. Se supplementation increased blood Se concentrations [30] and this increase may enhance the muscle GPx enzyme activity as it has been previously described in animal models [37] and in acute exercise in humans [30]. However, moderate- or low-endurance exercise did not change muscle GPx enzyme activity between the Se group and placebo group [13,30] and muscle GPx enzyme was not correlated with Se plasma levels [12]. It may be possible that the training intensity, volume, and the frequency of exercise [13,30] were sufficient to induce an upregulation muscle GPx activity to control the OS, but in acute exercise [30] Se supplementation is necessary for antioxidant GPx defense. This may suggest that exercise and Se are linked to erythrocyte GPx [26] or muscle GPx [30] activity, resulting in effects on the development of antioxidant potential, which could result in better protection of membranes at this level (muscle and/or erythrocyte). The intensification in muscle-cell membrane defense may lead lower exercise-induced muscle damage in athletes. Also, OS repercussions on the phospholipid bilayer repair and the integral proteins were associated to erythrocyte cytoskeleton, including Band 3 protein, and they participated in erythrocytes deformability and tissue oxygenation [38]. Therefore, the aforementioned antioxidant effect was a factor that improved the transport and use of oxygen at a muscular level, ehich is particularly important because endurance and aerobic capacity is key for atheltes. Athletes generally have a very demanding training. Intense anv vigorous training may improve sport performance in competitions [4,6]. The generation of radicals and other ROS during exercise in muscle, and the antioxidant defense provided by Se, may establish an interrelationship, in which they may play a key role of Se mineral trace element in exercise performance [35]. Theincrease in GPx in plasma [13,26], erythrocyte [26] or muscle [30] activity may be sensitive to the Se supplementation in the form of selenomethionine with doses between 180–240 µg/day.

In this systematic review, we observed the absence of effect of Se supplementation in other molecules related to the enzymatic defense system. This may be partially explained because the adaptive effects of exercise were adequate to induce these enzymes. For the technique of application to quantify total antioxidant status (TAS), Savory et al. [19] proposed a presumed “index of antioxidants”, or diminishing potentiality of biofluids. No differences in TAS were detected between groups, of the study at rest or post-exercise pre- and post-Se supplementation [19]. Although other studies included in this review did not evaluate TAS, we hypothesized that Se did not have had any influence in TAS. The potential explanation for this may rely in the effect of the Se supplementation was only on GPx activity, and overall the antioxidant activity could be the result of all the enzymes involved and not just the individual action of one enzyme. This may suggest that the protection of the cell has to be guaranteed by a synergistic action of all the antioxidant enzymes and not just only one. In addition, vitamin E plays an antioxidant role in the organism the differences were not detected between control and Se group [13,26,30].

It may result difficult to specify that Se supplementation antioxidant effect because the wide variation in OS reduction following Se supplementation which flow from divergences in study methodology. In addition, the concentration of seleoenzymes dependents on the amount of Se administered, which is prevalent due to the influence of seleno-protein expression. One more important factor is the inter-individual response of selenoenzymes to Se supplementation, which may indicate that the amount of Se should be tailored to each subject, even within the same population group [9,33,36].

### 4.3. Muscle Performance

CK activity assessed in blood samples is often used to monitor the levels of muscle damage in exercise [7]. One study [13] reported that CK activities did not significantly differ between pre- and post-training. The absence of change in CK after 200 µg Se (sodium selenite) supplementation may suggest a lack of an antioxidant function that is not able to neutralize ROS produced during the electron transport chain oxidative phosphorylation necessary for energy requirements in physical exercise [7].

The response of human skeletal muscle to endurance training may result of a transformation from histological type II fibers to type I fibers and may induce a decrease in the respiratory mitochondrial activity caused by the production of oxygen-free radicals [39]. In non-trained subjects after 10 weeks of endurance training and a 180 µg of Se (seleniomethionine) supplementation, no effects were observed on exercise training-induced adaptations on oxidative enzyme activities or on expressed muscle myosin heavy chain (MHC) fiber type. Therefore, it may be probably that Se supplementation may have no effect on exercise-induced muscle adaptations.

Some studies [21,39] have reported that exercise depressing mitochondrial respiratory capacity may occur due to the interruption of mitochondrial structure. These mithochondrial degradations are compensated by the development of antioxidant defense systems in the cytosol and the membranes such as GPx that is an essential component of the antioxidative capacity of muscle fibers. Zamora et al. [29] reported that 180 µg of Se (seleniomethionine) supplementation may indicate the cushioning effect of the Se on the mitochondria alteration (density of the mitochondria profile; surface of all the mitochondria profile areas). The observation of the muscle biopsies using the electron microscope confirmed these changes in the muscle mitochondria. In addition, quantitative determination of the totality of mitochondria in the muscle fibers was performed [29]. Although the mechanism by which mitochondrial turnover occurs is unknown, it may be related to the enhanced activity of the Se-dependent enzyme GPx. Some authors have reported that the activity of the muscle GPx enzyme increased during prolonged exercise after training with Se supplementation [26,30]. The GPx enzyme may seem to be a powerful component of the muscle antioxidant defense system. A potential mechanism for the suggested dampening effect of Se may rely in the mitochondria biogenesis, which may be a consequence of the increased activity of GPx, avoiding OS-induced cellular degeneration, which occurrs during continuous and strenuous mesocycles of training.

### 4.4. Hormone Response

The function of the hypothalamus-pituitary-adrenal axis increases the levels of plasma adrenocorticotropic hormone [7]. In general, continuous and intensive exercise has been reported to induce a dysfunction of the hypothalamic-pituitary-testicular axis, particularly testicular impairment. This dysfunction may cause a suppression of the testosterone (T) secretion during latter stages of exercise. The levels of T hormone is indicative of the degree of anabolism/catabolism of the body and can be used as a biomarker to monitor and optimize athletes’ training loads [40]. T metabolism and testicular morphology may explain the existence of other selenoproteins in the male gonads [3]. Neek et al. [18] observed that 200 µg/day (Sodium Selenite) of Se supplementation during 4 weeks had no effect on levels of total and free T at resting. These researchers additionally observed that there were no significant differences in T between intervention and placebo group in pre- and post-exercise. Therefore, Se supplementation did not seem to stimulate the activity of protection against GPx peroxidation (Se-dependent) that is required for the metabolic pathway of T biosynthesis in Leydig cells. T synthesis levels may change depending on the activity of this enzyme. Additionally, only one study [18] assessed the effects of Se supplementation on testosterone hormone response, therefore further studies are needed.

### 4.5. Athletic Performance

Regarding the Se supplementation and athletic performance, Neek et al. [18] found no differences between the control and intervention group among professional cyclists [18]. These findings are reported by other studies that demonstrated that intakes of 180 µg of Se (seleniomethionine) during 10 weeks of endurance training, in healthy students, had no effects on aerobic performance (VO_2_max) [13,26,29]. 

Some authors [13] have described correlations between the proportion of type I MHC and VO_2_max increments which may suggest a relationship between endurance performance capacity and muscle fiber type. Probably, the magnitude of adaptive responses may depend on the quantity of muscle contractile activity during exercise rather than the levels of Se. Other authors [26] have found significant increases in erythrocyte GPx when supplementing with Se and this may be explained the correlation between erythrocyte GPx/VO_2_max [26]. These results may suggest that the participation of physiological antioxidant potential in the mechanism of development of aerobic performance had no direct effect on VO_2_max, which only increased after exercise and has been reported previously [29].

Plasma lactate concentration increased with higher levels of exercise and may be useful for monitoring anaerobic training [41]. Se supplementation may boost the antioxidant system when exercising [9]. In animal models, the increase in free radical production and blood lactate concentrations due to acute swimming exercise may be compensate with Se supplementation [36]. However, in a study that included 16 professional cyclists, oral supplemented with 200 µg/day (sodium selenite) had no effects on plasma lactate in pre- and post- exercise [18]. For this reason, the Se supplementation may have no effect on anaerobic glucose metabolism.

### 4.6. Practical Applications

In general, the selected studies suggest that the use of Se as a supplement may decrease the lipid peroxidation generated by an intense and continuous physical activity. However, there was no evidence of improvement on athlete performance. Although Se supplementation did not demonstrate beneficial effects on athletic performance, Se supplementation may be required to maintain optimal levels of Se, and prevent negative health outcomes of the athletes [42]. In athletes, several etiological factors such as gastrointestinal loss, increased loss of Se through sweating and urine, intestinal malabsorption, and malnutrition may explain the storage depletion of Se, which may lead to deficiencies in Se [18,34]. Health problems related to Se deficiency have been described in athletes with vigorous and strenuous training [4]. Therefore, Se supplementation may be a simple approach to reduce chronic oxidative stress among individuals with Se deficiency [19]. Furthermore, the Se supplementation may be beneficial for athletes because it may reinforce the antioxidant potential activity by increasing the level of GPx in erythrocytes [26] or muscles [13,30], and increase plasma Se concentration [13,26,30]. Moreover, Se supplementation may prevent some diseases such as Keshan disease (KD), an endemic cardiomyopathy that is exclusively manifested in China among populations with Se deficiency [43]. Moreover, Se supplementation in athletes who usually perform high-intensity exercise training may prevent low levels of immune system, and consequently, decrease the risk of infection [44]. Therefore, Se might be hypothesized as a strategy to prevent emerging viral diseases, such as COVID-19. The immunomodulatory properties of Se, together with the ability to limit the mutation and progression of the virus, may suggest that optimal levels of Se in the blood may have positive effects against COVID-19 [45].

On the other hand, we believe that more studies performed among athletes are needed. Athletes may be very susceptible to Se defficiens because their vigorous and intense practices. Special caution should be noted when recommending Se supplementation. Elevated levels of Se may cause toxicityand induce excessive mitochondrial oxidative stress, leading to organelle damage and dysfunction [2]. In this review none of the selected studies [13,18,19,26,29,30] reported any side effects of Se supplementation among the participants. 

### 4.7. Limitations and Strengths

We acknowledge the following limitations. First, the systematic review included a small number of articles that were conducted quite a time before. In addition, all the studies had a small sample size (mostly men), and only 2 studies reported Se plasma concentrations. Second, the intervention characteristics of the studies such intensity and duration of exercise, timing and dose of Se supplementation, and the number of participants, among many others, were quite different. Consequently, it results difficult to draw strong conclusions on whether Se supplementation is recommended as a sport supplement. 

On the other hand, this systematic evidences the need of further studies to re-evaluate the effects of long periods of Se supplementation on antioxidant defense system, muscle performance, and hormonal response to determine potential improvements in sports performance. Despite these limitations, the strengths of this systematic review rely in the use of the PRISMA guidelines [27] the McMaster Quantitative Review Form [28].

## 5. Conclusions

In summary, we found no evidence of beneficial effects of the use of Se supplementation on aerobic or anaerobic athletic performance. However, Se supplementation may contribute to maintain optimal levels in athletes who have significant losses from high-intensity and high-volume exercise and, consequently, reduce chronic exercise-induced oxidative stress. Optimal levels of Se modulate exercise-effect on mitochondrial changes (structure, respiratory) probably because the high efficiency of the Se-dependent enzyme GPx that increased during prolonged exercise. Therefore, Se supplementation may be used as enhancer of antioxidant potential activity in physically active individuals.

## Figures and Tables

**Table 1:** Characteristics of participants and interventions in the studies included in the review.

**Level of Participants**	Active	1 Study [18]
	Regularly trained athletes	4 Studies [13,26,29,30]
	No Regular Training before the Study	1 Study [19]
**Age Range (years)**	20–35 years	5 Studies [13,19,26,29,30]
	Not Specified	1 Study [18]
**Se Plasma Level (µg/l)**	Assayed	4 Studies [13,19,26,30]
	Not Assayed	2 Studies [18,29]
**Type of Administration of Selenium**	Organic selenium in form of selenomethionine	4 Studies [13,26,29,30]
	Salts of sodium selenite (Na_2_Se0_3_)	2 Studies [18,19]
**Dosage Used**	180 µg single dose	3 Studies [13,26,29]
	200 µg single dose	2 Studies [18,19]
	240 µg single dose	1 Study [30]
**Moment of Supplementation**	Daily	6 Studies [13,18,19,26,29,30]
**Duration of Treatment**	4 weeks	1 Study [18]
	10 weeks	4 Studies [13,26,29,30]
	14 weeks	1 Study [19]

**Table 2:** Methodological quality of the studies included in the systematic review.

References		Margaritis et al. 1997 [13]	Zamora et al. 1995 [29]	Savory et al. 2012 [19]	Tessier et al. 1994 [26]	Neek et al. 2011 [18]	Tessier et al. 1995 [30]	T_I_
ITEMS	**1**	1	1	1	1	1	1	6
	**2**	1	1	1	1	1	1	6
	**3**	1	1	1	1	1	1	6
	**4**	1	1	1	1	1	1	6
	**5**	1	1	1	1	1	1	6
	**6**	0	0	1	0	0	0	2
	**7**	1	1	1	1	1	1	6
	**8**	1	1	1	1	1	1	6
	**9**	0	1	1	1	1	1	5
	**10**	0	0	0	0	0	0	1
	**11**	1	1	1	1	1	1	6
	**12**	1	1	1	1	1	1	6
	**13**	1	1	1	1	1	1	6
	**14**	0	0	0	0	0	0	0
	**15**	1	1	1	1	1	1	6
	**16**	1	1	1	1	1	1	6
T_S_		12	13	14	13	15	13	
%		75	81.3	87.5	81.3	93.8	81.3	
MQ		G	VG	VG	VG	E	VG	

(T_S_) Total items fulfilled by study. (1) Criterion met; (0) Criterion not met. (T_I_): Total items fulfilled by items. Methodological Quality (MQ): poor (P) ≤8 points; acceptable (A) 9–10 points; good (G) 11–12 points; very good (VG) 13–14 points; excellent (E) ≥15 points.

**Table:** (**A**)

Authors & Year	Study Design	Population	Intervention	Analyzed Results	Main Conclusions	
Savory et al., 2012 [19]	Placebo-controlled, double-blind, crossover	20 healthy subjects 9♂ & 1♀ NW: 4 ♂ & 6 ♀ 27.9 ± 2.2 y BMI 22.8 ± 0.4 kg/m^2^ OW: 5 ♂ & 5 ♀ 31.4 ± 1.9 y BMI 28.0 ± 0.8 kg/m^2^	Supplementation: 200 μg Se (sodium selenite) once per day * 3 weeks’ placebo (not containing glucose) during another 3-week period. Washout period 2 moth. Order treatment: NW: Se/Placebo OW: Placebo/Se 14-week total period PhA: test 30 min treadmill session at 70% VO_2_peak	[Se]	Post Se treatment period ↑ *[Se] NW & OW compared to week 0	
					Post Se treatment period ↑* [Se] NW & OW compared to placebo treatment	
				TAS, GSH, SOD	Placebo period & Se treatment period - rest vs. post PhA: OW & NW ↔ TAS; GSH; SOD - OW vs. NW ↔ TAS; GSH; SOD	
				LH	Placebo period - rest vs. post PhA: OW ↑*LH NW ↑LH - OW vs. NW ↑*LH	
					Se treatment period - rest vs. post PhA: OW †LH NW †LH − OW vs. NW †LH	
					Placebo vs. Se treatment post PhA: OW ↓*LH; NW↔LH	
Tessier et al., 1994 [26]	Placebo-controlled, double-blind, randomized	24 ♂ healthy students 22.9 ± 2.1 y; 8.0 ± 8.7 Kg; 178.0 ± 6.6 cm; Body fat 11.2 ± 4.4 % PbG *n =* 12 ♂ 22.5 ± 2.0 y; 67.3 ±7.0 Kg; 177.4 ± 7.0 cm; Body fat 10.4 ± 3.9 % SeG *n =* 12 ♂ 23.2 ± 2.3 y; 68.7 ± 10.4 Kg; 178.7 ± 6.3 cm; Body fat 12.3 ± 4.8 %	Supplementation: 180 μg Se (Seleniomethionine) once per day * 10-week period PhA: 10-week endurance training program 4-week nontraining		Pre-PhA vs. Post-PhA	SeG vs. PbG
				[Se]	↑*[Se] SeG ↓[Se] PbG	# [Se]
				GTtotal	↓* SeGr ↓* PbGr	† GTtotal
				GSSG	↓ GSSG SeGr ↓ GSSG PbGr	† GSSG
				GPx plasma	↑* SeG ↑PbG	# GPx
				EGPx	↑* SeG ↑PbG	# EGPx
				EGR	↑* SeG ↑*PbG	† EGR
				Vitamin E	↓SeG ↑PbG	† Vitamin E
				VO2max	↑* SeG ↑*PbG	† VO2max
					SeG: ↑ VO2max positive correlated ↑GPX (r:0.66 *p* < 0.05 *n =* 12)	

**Table:** (**B**)

Author/s—Year	Study Design	Population	Intervention	Analyzed Results	Results and Main Conclusions	
Neek et al., 2011 [18]	Placebo-controlled, double-blind, randomized	16 ♂ road cyclists Pb *n =* 8 ♂ cyclists 66.1 ± 4.2 Kg; 176.8 ± 8.0 cm; BMI 21.1 ± 4.4 Kg/m^2^ SeG *n =* 8 ♂ cyclists 61.6 ± 4.7 Kg; 177.7 ± 4.2 cm; BMI 20.5 ± 1.2 Kg/m^2^	Supplementation: 200 μg de Selenium (sodium selenite) once per day * 4-week period PhA: cycling exhaustive exercise 4-week		Pre-PhA vs. Post-PhA	SeG vs. PbG
				Tt	↑* SeG ↑* PbG	↔ Tt
				Tf	↑* SeG ↑* PbG	↔ Tf
				[La]	↑* SeG ↑* PbG	↔ [La]
Margaritis et al., 1997 [13]	Placebo-controlled, double-blind, randomized	24 ♂ healthy subjects 22.9 ± 2.2 y; 68.0 ± 8.7 Kg; 178.1 ± 6.6 cm; Body fat 11.2 ± 4.9 % PbG *n =* 12 ♂ 22.5 ± 2.0 y; 67.3 ± 7.0 Kg; 177.4 ± 7.0 cm; Body fat 10.1 ± 4.0% SeG *n =* 12 ♂ 23.3 ± 2.4 y; 68.8 ± 10.4 kg; 178.8 ± 6.4 cm; Body fat 12.6 ± 4.9 %	Supplementation: 180 μg Se (Seleniomethionine) once per day * 10-week period PhA: 10-wk endurance training program, 3 sessions per week		Pre-PhA vs. Post-PhA	SeG vs. PbG
				[Se]	↑*[Se] SeG ↓[Se] PbG	# SeG vs. PbG
				GPx plasma	↑*SeG ↑PbG	# SeG vs. PbG
				GPx muscle	↓ SeG ↓ PbG	† SeG vs. PbG
				Vitamin E	↓ SeG ↑PbG	† SeG vs. PbG
				CK	↓ SeG ↓PbG	† SeG vs. PbG
				Cyt Ox	↑ SeG ↑PbG	# SeG vs. PbG
				SDH	↑ SeG ↑ PbG	† SeG vs. PbG
				MHC I	↑SeG ↑PbG	# SeG vs. PbG
				MHC II	↓SeG ↓PbG	† SeG vs. PbG
				MHC I - MHC II co-expressed	↑SeG ↑PbG	# SeG vs. PbG
				VO_2_max	↑*SeG ↑*PbG	# SeG vs. PbG
				VO_2_total	↑*SeG ↑*PbG	# SeG vs. PbG

**Table:** (**C**)

Author/s—Year	Study Design	Population	Intervention	Analyzed Results	Main Conclusions			
Zamora et al., 1995 [29]	Placebo-controlled, double-blind, randomized	24 ♂ healthy students; 22.9 ± 2.1 y; 68.0 ± 8.7 Kg; 178.1 ± 6.6 cm; Body fat 11.2 ± 4.4 % PbG *n =* 12 ♂ 22.5 ± 2.0 y; 67.3 ± 7.0 Kg; 177.4 ± 7.0 cm; Body fat 10.1 ± 3.9 % SeG *n =* 12 ♂ 23.28 ± 2.36 y; 68.78 ± 10.44 Kg; 178.7 ± 6.3 cm; Body fat 12.3 ± 4.8 %	Supplementation: 180 μg Se (Seleniomethionine) once per day * 10-week period PhA: 10-week endurance training programme (3 sessions per week) after a 4-week period of restricted		At rest		Post-PhA	
					Pre-PhA vs. Post-PhA	SeG vs. PbG	Pre-PhA vs. Post-PhA	SeG vs. PbG
				Muscle mitochondria morphometric parameters Q_A_ A_a_ â	↑*SelG ↔ PbG ↑*SelGr ↔ PbG ↔ SelGr ↔ PbG	#SelGr vs PbG #SelGr vs PbG #SelGr vs PbG	↑*SelG ↔ PbG ↑*SelG ↔ PbG ↔ SelGr ↔ PbG	#SelGr vs PbG ↔ SelGr vs PbG #SelGr vs PbG
				VO_2_max	↔ SelGr ↔ PbG	† SelGr vs PbG	↔ SelGr ↔ PbG	† SelGr vs PbG
				Body fat %	↔ SelGr ↔ PbG	† SelGr vs PbG	↔ SelGr ↔ PbG	† SelGr vs PbG
				BMI Kg*m^2^	↔ SelGr ↔ PbG	† SelGr vs PbG	↔ SelGr ↔ PbG	† SelGr vs PbG
Tessier et al., 1995 [30]	Placebo-controlled, double-blind, randomized	24 ♂ healthy; 22.9 ± 2.1 y	Supplementation: 240 μg organic selenium (70% selenomethionine) Selenion^®^ once per day *10-week period PhA: 4-week deconditioning period with no training, followed by running endurance training lasting 10 week (3 sessions/week).		Pre-PhA vs. Post-PhA		SeG vs. PbG	
				[Se]	↑*SelG ↓PbG		† SelGr vs PbG	
				Vitamin E	↓SelG ↑PbG		† SelGr vs PbG	
				GPx muscle				
				PhA cronic	↓SelG ↓PbG		† SelGr vs PbG	
				PhA acute	↑*SelG ↓*PbG		# SelGr vs PbG	

♂: Men; ♀: Women; NW: Normal weight; OW: Over weight; y: Years; Kg: Kilograms; m: Meters; cm: Centimeters; BMI: Body mass index; PhA: Physical activity; Se: Selenium; [Se]: Plasma Selenium levels; ↑*: Statistically significant increase; ↑: Non-statistical increase; ↓*: Statistically significant decrease; ↓: Non-statistical decrease; †: Change without statistical significance; #: Change statistical significance; ↔: Without changes; LH: Lipid hydroperoxidase; GSH: Reduced glutathione; SOD: Superoxide dismutase; TAS: Total antioxidant status; PbG: Placebo group; SeG: Selenium group; GT_total_: Glutathione total; GSSG: Glutathione oxidized; GPx: Glutathione peroxidase; EGPx: Erythrocyte glutathione peroxidase; EGR: Erythrocyte glutathione reductase; VO_2_max: Maximum oxygen consumption; Tt: Total testosterone; Tf: Testosterone free; [La]: Plasma lactate; CK: Creatine kinase; Cyt Ox: Cytochrome C oxidase; SDH: Succinate dehydrogenase; MCH: Myosin heavy chains; Q_A_: Density of the mitochondria profile; A_a_: surface of all the mitochondria profile area; â: mean surface of individual mitochondria profile area.
